# Clinical Lecture, Gynæcological Department, Cook County Hospital

**Published:** 1874-11-15

**Authors:** T. Davis Fitch


### CLINICAL LECTURE, GYNÆCOLOGICAL DEPARTMENT COOK COUNTY HOSPITAL.

### Service of Prof. T. Davis Fitch.

*Reported by D. A. K. Steele, Resident Physician.*

GENTLEMEN: To-day we will discuss Vaginitis, or, an inflammation of the mucous membrane lining the vaginal canal. Several varieties of vaginitis are recognized as acute. The latter of the following varieties: simple, specific, and granular, or glandular. Other varieties are mentioned by some authors, such as puerperal, by Prof. Byford, of this city, and diphtheritic, by Scanzoni, &c., &c.

Acute vaginitis may be simple or specific as regards its origin.

The simple form begins usually with swelling of the vulva, and heat, burning itching, and pains at vulvar extremity of the vagina, often around urethra; and one of the first symptoms complained of will be painful micturition. The inflammation may extend to all mucous canals along the urethra, lighting up a urethritis, perhaps to the bladder, giving a cystitis; along the vagina to cervix, giving an endo-cervicitis; or to the cavity of the uterus, giving rise to an endo-metritis; or even along the fallopian tubes to the peritoneal cavity, kindling a fatal peritonitis.

The inflammation may be primary or secondary: primary where it originates in the vaginal mucous membrane; secondary where it proceeds from a vulvitis, or is caused by the irritation of the discharge from an endo-cervical inflammation. In an acute vaginitis, for the first forty-eight hours, you have increased heat and dryness of the parts, succeeded by a copious muco-purulent discharge, of an ichorous nature, which in a few days becomes yellowish, and perhaps assumes a greenish hue; this discharge is continuous, and might be confounded with an endo-metritic discharge, but in the latter you would have it tinged with blood. You might also confound it with an old pelvic cellulitis, when there was a sinus existing, but you would readily be able to differentiate by means of a specular examination, and the history of the case.

The physical signs of acute simple vaginitis, are swelling of the vulva, contraction of the vagina, and you will see the mucous membrane of a deep red color, sometimes almost scarlet, differing essentially from the normal vaginal mucous membrane, which should have a pinkish hue, resembling exactly the buccal mucous membrane.

The specific variety, or gonorrhœal vaginitis, differs very slightly from the simple. Very often you cannot tell any difference. Some authorities distinguish them by reason of the greater acuity of the symptoms manifested in the latter variety; the increased urethral inflammation; greater redness and pain in the parts; the mucous surface bleeding freely and being more sensitive on examination; but, gentlemen, be exceedingly careful in giving a positive diagnosis, for the two varieties resemble each other so closely that you will often not be able to detect the slightest difference, and by giving a hasty opinion you may do irreparable injury to an innocent party, and inflict a sting that you can never extract; for if once your fiat has gone forth you will find that by no amount of argumentation can you change the impression or suspicion you have created.

I would by no means advise you to be dishonest with your patients, but, by the exercise of a little *finesse*, you may retain their confidence and friendship when suffering from this affection, when, otherwise, you might cause infinite family trouble and disturbance. I recall a number of incidents of this nature, in my own practice, where a little imprudence in the management of these cases would have disrupted families.

A man may contract a urethritis from the acrid vaginal discharge, occurring during menstruation, or from an endo-cervical discharge.

There is another variety of vaginitis, termed the follicular, generally the result of pregnancy, in which we have the mucous membrane studded with little red points, that are the hyperæmic and hypertrophied papillæ. This variety may result from either of the other forms.

Then we have the chronic, which is simply a continuation of the acute, in which the severity of the symptoms is modified. The ordinary duration of an acute vaginitis is about two weeks; that of the chronic form is indefinite; may run on for a number of weeks, months, and occasionally lasts for a number of years. I have a patient now under treatment for some two years, with but little benefit. As regards treatment, cure the cause in the first place. If due to an endo-metritis, or a vulvitis, address your remedies to the cure of the primary affection.

In the acute form use warm sitz baths, mucilaginous vaginal injections, and give saline laxatives; if pain is severe give anodynes. A very excellent formula for a vaginal injection, and the one that I am in the habit of prescribing, is

Pot. chloras, ʒ iv.

Pot. permang. gr. x.

Aquæ, ℥ xvj. M.

A teacupful morning and evening, with a little warm water added.

Another very excellent plan is the use of cotton tampons of glycerite of tannic acid.

Introducing one of these tampons every three or four days, by attaching a string to them the patient can very readily remove them herself. In using them you should be careful to caution the patient about the liability of staining her clothing from the tannin, in order that she may wear an appropriate bandage.

